# The effect of acute vs chronic magnesium supplementation on exercise and recovery on resistance exercise, blood pressure and total peripheral resistance on normotensive adults

**DOI:** 10.1186/s12970-015-0081-z

**Published:** 2015-04-24

**Authors:** Lindsy S Kass, Filipe Poeira

**Affiliations:** University of Hertfordshire, School of Life and Medical Science, College Lane, Hatfield, Hertfordshire AL10 9AB UK

**Keywords:** Magnesium supplementation, Blood pressure, Bench press, Acute and chronic loading

## Abstract

**Background:**

Magnesium supplementation has previously shown reductions in blood pressure of up to 12 mmHg. A positive relationship between magnesium supplementation and performance gains in resistance exercise has also been seen. However, no previous studies have investigated loading strategies to optimise response. The aim of this study was to assess the effect of oral magnesium supplementation on resistance exercise and vascular response after intense exercise for an acute and chronic loading strategy on a 2-day repeat protocol.

**Methods:**

The study was a randomised, double-blind, cross-over design, placebo controlled 2 day repeat measure protocol (n = 13). Intense exercise (40 km time trial) was followed by bench press at 80% 1RM to exhaustion, with blood pressure and total peripheral resistance (TPR) recorded. 300 mg/d elemental magnesium was supplemented for either a 1 (A) or 4 (Chr) week loading strategy. Food diaries were recorded.

**Results:**

Dietary magnesium intake was above the Reference Nutrient Intake (RNI) for all groups. Bench press showed a significant increase of 17.7% (p = 0.031) for A on day 1. On day 2 A showed no decrease in performance whilst Chr showed a 32.1% decrease. On day 2 post-exercise systolic blood pressure (SBP) was significantly lower in both A (p = 0.0.47) and Chr (p = 0.016) groups. Diastolic blood pressure (DBP) showed significant decreases on day 2 solely for A (p = 0.047) with no changes in the Chr. TPR reduced for A on days 1 and 2 (p = 0.031) with Chr showing an increase on day 1 (p = 0.008) and no change on day 2.

**Conclusion:**

There was no cumulative effect of Chr supplementation compared to A. A group showed improvement for bench press concurring with previous research which was not seen in Chr. On day 2 A showed a small non-significant increase but not a decrement as expected with Chr showing a decrease. DBP showed reductions in both Chr and A loading, agreeing with previous literature. This is suggestive of a different mechanism for BP reduction than for muscular strength. TPR showed greater reductions with A than Chr, which would not be expected as both interventions had reductions in BP, which is associated with TPR.

## Background

Magnesium (Mg^2+^) elicits significant enzymatic cellular involvement and physical regulation such as energy metabolism/production through formation of the Mg-ATP complex [1] and physiological regulation and control of neuromuscular cardiac activity, muscular contraction, vascular tone and blood pressure [2,3]. Its effect on muscular contraction and vascular tone have been shown to reduce blood pressure and subsequently vascular resistance [4].

Nutritional supplementation is a well-established method for enhancing performance in conjunction to training. Micronutrient intake has been highlighted to have gained greater prominence with athletes in relation to the importance of an adequate nutritional status [5], However, previous research highlights nutritional inadequacies and thus an impaired nutritional status (i.e. marginal nutrient deficiency) from both an athletic [5] and general population perspective [6]. This identifies physical activity as increasing the rate at which micronutrients are utilised, promoting excessive micronutrient loss via increased catabolism and excretion (sweat and urine). Magnesium is a mineral required at rest and during exercise [7]. This increase in Mg^2+^ turnover during exercise may lead to a state of insufficiency acting as a contributory factor towards an increase in blood pressure and a state of hypertension [8]. This, together with a decline in dietary intake below the RNI may have a negative impact on both performance and blood pressure.

Magnesium supplementation in relation to exercise has differed considerably in research opinion as to the dose and type of Mg2+ salt administered. It is influenced by the specific anion attachment with Mg2+, thus influencing supplemental solubility, elemental Mg2+ bioavailability and supplemental effectiveness [9]. Research has illustrated organic forms of Mg2+ supplementation i.e. Aspartate, citrate, lactate, pidolate, fumurate, acetate, ascorbate and gluconate to exemplify a greater solubility and bioavailability in comparison to inorganic forms i.e. oxide, sulphate, chloride and carbonate [10] When considered relative to the quantity needed to be ingested to release 300 mg of elemental Mg2+ along with the fact that certain magnesiums are unavailable in the UK, magnesium citrate was considered to be the best option for this protocol.

Research to date consists of both positive [11-13] and negative [14,15] findings. The research appears to agree that Mg^2+^ supplementation has no effect on physical performance when serum concentrations are within the normal range (serum Mg^2+^ 0.8-1.2 mmol·L^−1^) [12,16]. However, manipulating intakes of Mg^2+^ by diet or supplementation has been shown to have performance [11,17] and blood pressure enhancements [13,18]. Limitations to many of these studies is the lack of information regarding either serum magnesium or dietary intake [19]. The general consensus appears to be that Mg^2+^ supplementation has a greater effect when habitual dietary intake or serum levels are low.

Further, to the best of the authors’ knowledge research to date lacks analysis of Mg^2+^ from an acute (A) and chronic (Chr) viewpoint within the same study. Therefore, the aim of the current study was to assess the effect of oral Mg^2+^ supplementation on strength performance and vascular responses from both an A and Chr loading strategy as to establish potential differences in supplemental duration and influences of dietary status and supplemental dose on performance and vascular responses.

## Methods

### Subjects

A total of 13 subjects (males (m) = 7 females (f) = 6) were recruited from recreational running, cycling and triathlete clubs. Six subjects were allocated randomly to the acute intervention group (m = 3, f = 3) and 7 to the chronic intervention group (m = 4, f = 3). Subjects were recruited in accordance to meeting the inclusion/exclusion criteria, (Table 1). Informed consent and health screen were completed and ethical approval was granted by the University of Hertfordshire School of Life Sciences Ethics Committee.

**Table 1:** **Subject characteristics; including group sample size (n), age, height, weight, VO**_**2max**_ **, HR**_**max**_

	**Chronic**	**Acute**
**N**	**7**	**6**
Age (years)	40.8 ± 4.4	35.8 ± 6.2
Height (cm)	176.2 ± 11	174.6 ± 12
Weight (kg)	73.2 ± 13.2	72.1 ± 13
VO_2_max (ml/kg)	51.8 ± 9.1	53 ± 4.8
HRmax (bpm)	176.4 ± 3.8	180.8 ± 7.7

Values are mean ± SD.

### Experimental design

The study was a randomised, cross-over, double-blind, placebo controlled, 2 day repeated measure protocol. Subjects were assigned to either the acute or chronic intervention and the two trials ran parallel. Within each trial subjects undertook both the magnesium intervention and a placebo intervention with a one week washout period in a randomised order. The two interventions were a chronic (Chr) (4 weeks) and acute (A) (1 week) loading strategy, sub-divided into a supplemental and a placebo control group with a 1 week washout period. A maximal graded exercise test for determination of VO_2max_ was conducted to ensure participant homogeneity with a cut off of 45 ml/kg^−1^ and 35 ml/kg^−1^ oxygen for males and females respectively. The study was tested across 2 consecutive days at each treatment time-point i.e. baseline and again after either 1 or 4 weeks intervention. A one week washout was then given and then a further intervention of the opposite treatment was given (placebo or magnesium) with the same loading phase.

### Protocol

After familiarisation, subjects were tested for baseline measurements Anthropometric measures (height (cm), weight (kg)) and age (y)) were recorded. All subjects attended a familiarisation session on all equipment and testing protocols prior to testing. On both day 1 and recovery day 2 participants completed a 40 km time trial on bicycles owned by the subjects and set onto a rig. A set 40 km flat course with no wind setting was used on a Computrainer Pro ergometer (Computrainer, Seattle). All on-screen course data information was blinded, verbal encouragement was not given during the exercise testing. The time trial was carried out to elicit physiological stress as normally determined by training and competition. After a 30 minute rest participants completed the following tests to determine the effect of magnesium on strength and cardiovascular parameters.

Blood pressure, and augmentation index (Aix) were recorded at rest immediately and before the bench press. Subjects then performed a bench press corresponding to a 5 repetition maximum (5-RM) protocol [20]) for determination of their 1-RM. Upon completion, a 5-minutes rest period was given. Subsequently, a bench press at 80% 1-RM was performed to exhaustion. A measure of force (Newtons) was recorded during the bench press, with additional measures of blood pressure and Aix immediately upon completion of the bench press.

### Supplementation

Magnesium citrate and placebo (cornflour) were capsulated into large vegetarian capsules. Capsules consisted of a total of 75 mg of elemental Mg^2+^ citrate, (Pioneer analytical balance. OHAUS, UK), 4 capsules per day were taken orally, equating supplemental Mg^2+^ to a total daily dose of 300 mg/d elemental Mg^2+^. Supplements were ingested evenly throughout the day on a non-testing day, or ingested 3 hours before exercise testing. Finally, the supplementation period for both placebo and Mg^2+^ accounted for a total ingestion period of 1 week or 4 weeks within the A and Chr groups, respectively.

### Diet

A 4-day weighed food and beverage diary was recorded in relation to 3 weekdays and 1 weekend day, which was used for analysis of habitual dietary magnesium intake through use of dietary analysis software (Dietplan 6.70 Forestfield Software, UK).

### Statistical analysis

Data were analysed using SPSS version 20 (IBM limited, UK) and Microsoft excel 2007 for Windows. Box-whisker plots measured normality/data distribution and showed that the data were not normally distributed. Therefore non-parametric Wilcoxon 2 related samples tests were carried out on all results to look for differences. Alpha value was set at 0.05.

## Results

There were no statistically significant difference found between anthropometric data, VO_2max_ and HR determining a homogeneous cohort (Table 1).

Table 2 shows averaged dietary data for both the Chr and A groups. Both the Chr and A control groups showed no significant difference between macronutrient and magnesium ingestion.

**Table 2:** **Dietary intake, values are mean ± SD**

	**Chronic intervention**	**Acute intervention**	**Chronic placebo control**	**Acute placebo control**
Kcal	2513 ± 1201	2686 ± 938	3985 ± 519	3785 ± 734
CHO (g)	274 ± 170	296 ± 118	397 ± 209	343 ± 79
Fat (g)	96 ± 58	115 ± 49	114 ± 63	105 ± 48
Pro (g)	119 ± 38	114 ± 37	136 ± 66	129 ± 16
Mg^2+^ (mg)	375 ± 104	368 ± 173	551 ± 347	378 ± 79

### Performance

#### Bench press

**Figure 1:**
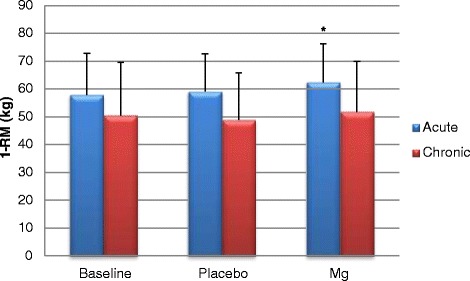
Acute and chronic bench press 1-RM scores on day 1 ± SD. * denotes significance.

Net strength gains as determined by 1-RM showed significant increase of 17.7% with the acute Mg^2+^ loading strategy compared to baseline (p = 0.031) (Figure 1). No significant strength gains were seen in the Chr intervention group (p = 0.281).

**Figure 2:**
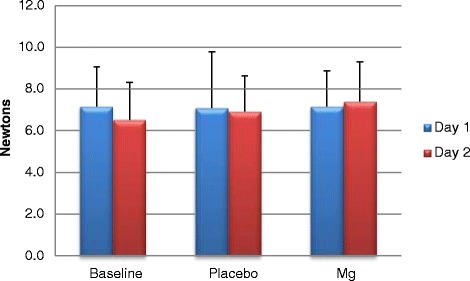
Acute force (newtons) output on day 1 and 2 (recovery) during repetitions to fatigue ± SD.

**Figure 3:**
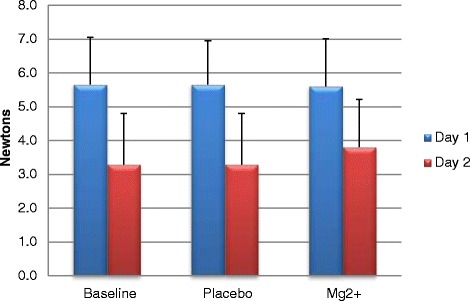
Chronic force (newtons) output on day 1 and 2 (recovery) during repetitions to fatigue ± SD.

Furthermore, A Mg^2+^ showed no decline in recovery (day 2) performance for force (N) resulting in a small day 2 (recovery day) force increase of 2.7%, showing a trend but no significnace (Figure 2). On the contrary Chr Mg^2+^ showed a day-to-day 32.1% performance decrement (Figure 3).

Resting SBP measures from day 1 and 2 show a significant decrease within A Mg^2+^ treatment (P = 0.031), conversely placebo showed a significant increase in SBP (P = 0.047) (Table 3). Further, significant day 2 reductions in SBP were noted between A treatments of Mg^2+^-placebo (P = 0.016). On the contrary, Chr Mg^2+^ shows no significant reductions in resting SBP on day 1 or day 2.

**Table 3:** **Acute and chronic group mean SBP values at rest and post bench press at 80% 1-RM to fatigue on day 1 and 2 ±SD**

**Physiological variable**	**Treatment**	**Group**		**Physiological variable**	**Treatment**	**Group**	
		**C Chr**	**A**			**Chr**	**A**
Resting SBP (mmHg) day 1	Placebo	119 ± 7	120 ± 5*^1^	Post SBP (mmHg) day 1	Placebo	143 ± 7*^3^	136 ± 5
	Mg2+	118 ± 6	122 ± 4*^2^		Mg2+	136 ± 9*^3^	137 ± 6
Resting SBP (mmHg) day 2	Placebo	121 ± 8	125 ± 2*^1, 3^	Post SBP (mmHg) day 2	Placebo	144 ± 9*^4^	144 ± 7*^5^
	Mg2+	118 ± 7	117 ± 7*^2, 3^		Mg2+	137 ± 10*^4^	134 ± 5*^5^

*Denotes significance as paired by numbers.

In relation to post SBP responses, both Chr and A Mg^2+^ treatment resulted in significant SBP reductions; however, such reductions can be noted on day 1 (P = 0.016) and day 2 (P = 0.016) for a Chr Mg^2+^ induced SBP reduction in comparison to placebo, whereas an A Mg^2+^ reduction can be accounted for on day 2 (P = 0.047) in comparison to placebo.

Resting DBP showed no difference for day 1 to day 2 between the placebo or Mg^2+.^Post DBP showed no differences between Day 1 to Day 2 for acute supplementation group (Table 4). However, chronic intervention showed a decrease in DBP for post bench press on the recovery day 2.

**Table 4:** **Acute and chronic group mean DBP values at rest and post bench press at 80% 1-RM to fatigue on day 1 and 2 ± SD**

**Physiological variable**	**Treatment**	**Group**		**Physiological variable**	**Treatment**	**Group**	
		**Chr**	**A**			**Chr**	**A**
Resting DBP (mmHg) day 1	Placebo	85 ± 7	75 ± 7	Post DBP (mmHg) day 1	Placebo	92 ± 8	85 ± 12
	Mg2+	79 ± 6	75 ± 4		Mg2+	87 ± 7	82 ± 5
Resting DBP (mmHg) day 2	Placebo	78 ± 8	79 ± 6	Post DBP (mmHg) day 2	Placebo	91 ± 5*^1, 2^	86 ± 13
	Mg2+	75 ± 7*^1^	74 ± 5*^2^		Mg2+	84 ± 8*^2^	76 ± 8*^3^

*Denotes significance as paired by numbers.

Although no significance was seen for Aix at rest for both A and Chr loading strategies, a significant lowering post bench press was found as highlighted in Table 5 on day 1 for A treatment and day 2 for the Chr treatment group. Day 1 Aix reductions correspond to a significant Mg^2+^ lowering effect compared to baseline (P = 0.016) and placebo (P = 0.031), respectively. Whereas, similar Aix reductions for the Chr Mg^2+^ group is noted on day 2 post bench press resulting in significant values of P = 0.039, when compared to baseline and placebo, respectively.

**Table 5:** **Acute and chronic group mean Aix values post bench press at 80% of 1-RM to fatigue on day 1 and 2 ± SD**

**Physiological variable**	**Treatment**	**Group**	
		**Chr**	**A**
Post Aix day 1 (%)	Baseline	7 ± 11	17 ± 5*^3^
	Placebo	9 ± 6	14 ± 6*^4^
	Mg2+	7 ± 5	10 ± 5*^3^ *^4^
Post Aix day 2 (%)	Baseline	14 ± 7*^1^	12 ± 6
	Placebo	14 ± 8*^2^	16 ± 4
	Mg2+	8 ± 12*^1, 2^	11 ± 6

*Denotes significance as paired by numbers.

## Discussion

This study set out to determine whether either acute or chronic magnesium supplementation would have an effect on performance (strength and cardiovascular) and blood pressure with exercise and/or on a second bout of exercise after a 24 hr recovery period. As has been shown previously [13,21] acute magnesium supplementation has a positive effect on BP, plyometric parameters and torque, however its effect on resistance exercise has not been evident to date. Further, chronic loading strategies have not been investigated in respect to exercise as well as the effect of Mg supplementation on a second bout of exercise. It was hypothesised that as acute Mg^2+^ supplementation has been seen to have beneficial effects on BP, CV parameters and peak torque a longer loading strategy (4 weeks) would amplify these results, giving a more beneficial and greater response. However, this study did not find that chronic loading of Mg^2+^ has a cumulative effect on the effect of supplementation, perhaps due to saturation of Mg^2+^ within the blood or limitations to transporters.

Primary findings showed variance across treatment groups on exercise (strength and recovery) and cardiovascular responses. The Chr Mg^2+^ intervention showed no significance in performance gains for bench press net strength and force output (Figures 1, 2 and 3). The A Mg^2+^ intervention showed variance in results across all variable analysed with some improvements being seen in resting HR and blood pressure for both Chr and A treatment groups regarding strength related performance (Figures 1, 2, 3 and Tables 3, 4, 5).

### Strength performance

Strength related performance within the bench press showed statistical significant improvements (P = 0.031) within the A group and Chr group. Previous research has shown that Mg^2+^ significantly enhances bench press [22] and strength performance [11,23]. Acute Mg^2+^ loading showed a significant net strength increase of 5.5 kg between baseline and supplemental Mg^2+^ trials. Other strength related measurements of force (Newtons) illustrated A Mg^2+^ induced improvements. Typically, where a decrease in force would be expected on day 2 (recovery) of training as a normal physiological response to training, an A group improvement of 0.25 Newtons (2.7%) was seen with Mg^2+^ supplementation compared to the Chr where a 2.0 Newtons (32.1%) decrement was seen.

When examining net strength of Chr compared to A groups a notable difference between baseline scores is evident implying that subjects within the A group might well be stronger due to a 7.3 kg 1-RM difference at baseline (Figure 1). Therefore, when considering the 10.5 kg difference between Chr and A group 1-RM trials after intervention of Mg^2+^, inter-subject lifting capacity/ability could be a factor of concern for validating such a difference.

These performance enhancements for the strength associated tests are suggestive of physiological-regulatory functions of Mg^2+^ within muscle contraction and relaxation; i.e. regulating troponin expression via Ca^2+^ concentration gradients, Ca^2+^ transport, MgATP complex formation optimising energy metabolism/muscular contraction, increasing protein synthetic rate, protection against cellular damage and, greater amount of actin-mysoin crossbridges [23-26] all of which contribute to the result of increased strength and force production. Consideration must be given as to why such a contrasting difference between Chr and A groups occur specifically when regarding strength performance measures. The A Mg^2+^ supplemented group showed day-to-day performance improvements across 3 trials, as opposed to 3 day-to-day non-performance improved trials exhibited within the Chr Mg^2+^ supplemented group which may be attributed to the different loading strategies within the current study. The Mg^2+^ supplementation within the current research was 300 mg/d, therefore equating the Chr and A group mean daily intake for Mg^2+^ to 675 mg/d and 700 mg/d, respectively, when combined with dietary Mg^2+^ intake as analysed from food diaries. This adds a sense of greater ambiguity when considering the Mg^2+^ - strength performance relationship, and comparing to research highlighting observations that intakes of 500 mg/d or greater result in further increases in strength [24,25]. It could be suggested that subjects within the Chr loading group might be more susceptible to a possible reduction threshold or cell tolerance for Mg^2+^ absorption based upon the understanding that high Mg^2+^ intakes result in a lower Mg^2+^ absorption [27]. Additionally, Mg^2+^ homeostasis may be postulated to exhibit no greater benefit from the chronic perspective due to the kidney function for Mg^2+^ excretion as to maintain a balanced concentration of Mg^2+^ [27,28]; for example, could the principle of a higher Mg^2+^ dose, longer supplemental duration and associated proportional increase of Mg^2+^ excretion highlight the body’s efficiency in maintaining a state of homeostasis? Alternatively, chronic loading through providing a regular high Mg^2+^ intake may influence extracellular Mg^2+^ concentrations which coincide with manipulation of Mg^2+^ transporter TRPM6 function, resulting in a potential decrease in TRPM6 expression in conjunction to increasing the urinary excretion of Mg^2+^ [29]. Thus, an acute ingestion rate as opposed to chronic could result in a more efficient use for Mg^2+^.

### Cardiovascular responses at rest and post bench press performance

Significant reductions in SBP and DBP are illustrated from post testing in the chronic group and rest and post testing in the acute group data across day 1 and 2 compared to baseline and placebo (Tables 3 and 4). Resting SBP was accounted for by a greater reduction in the A Mg^2+^ of 2 mmHg, in comparison to 0.7 mmHg with the Chr Mg^2+^ treatment. In addition, both resting and post DBP showed reductions with a greater day-to-day DBP reduction in the A Mg^2+^ in comparison to Chr Mg^2+^ as shown by a 69.2% and 50% (9 mmHg and 3 mmHg difference) at rest and post exercise for A and Chr groups respectively. These findings are in agreement with previous research [13,30] showing the importance of Mg^2+^ and its influence on blood pressure regulation. This is supported by findings within a recent meta-analysis [19] looking at Mg^2+^ supplementation which showed that SBP and DBP reductions of 2–3 mmHg and 3–4 mmHg, respectively. These observations oppose some previous findings which emphasise supplemental ineffectiveness of Mg^2+^ [31-33].

Such reductions in blood pressure could be speculated as being an outcome influenced by increases within the extracellular concentration of Mg^2+^, an effect that has been associated with reductions in the arterial tension and tone. These reductions in arterial tension and tone correspond to typical Mg^2+^ induced vasodilatory actions which potentiate effects of endogenous vasodilators such as adenosine, K^+^, nitric oxide and cyclo-oxygenase-dependent mechanisms via production of PGI2 [34]. In combination, Mg^2+^ acts as an antagonist to blocking Ca^2+^ channels [11,35,36] and further enzymatic mobilisation of Ca^2+^ [37]. Thus, data within the current study concur with previous research on the efficacy of Mg^2+^ supplementation in reducing blood pressure [13,38] and its capacity to suppress agonist vasoconstriction [4]. The above mechanisms may also be attributed to Mg^2+^ induced specific alterations within the vasculature, for example, Mg^2+^’s mediatory role within the endothelium corresponds to increased nitric oxide, PGI2 and decreases platelet aggregation, in combination to stringent down-regulation of Ca^2+^ voltage operated channel activity and release from the sarcoplasmic reticulum [39].

Average dietary Mg^2+^ intakes within the A and Chr groups corresponded to 368 mg/d and 375 mg/d, respectively. However, it must be considered that the blood pressure reduction in Chr and A loading strategies, may be attributed to the Mg^2+^ supplementation. With this in mind, it could be suggested that despite average dietary intakes of Mg^2+^ meeting the UK RNI a higher requirement for Mg^2+^ may be beneficial in reducing blood pressure. Further recommendations within the U.S are 420 mg/d and 320 mg/d for males, and females, respectively, in addition to Mg^2+^ requirements within the UK being determined many years ago [40]; Research by Geleijnse et al. [41] in a comparative study between 5 European countries which included the UK, corroborates with this study suggesting a potential increase of Mg^2+^ based on supplemental blood pressure enhancements, whereby the researchers highlighted a <350 mg/d of Mg^2+^ as suboptimal, augmenting the prevalence of hypertension. The study further accounted for an 80% insufficiency corresponding to Mg^2+^ intake to be evident within the UK population analysed [41].

A principle limitation within the current study concerns lack of monitoring of the subjects’ Mg^2+^ status via serum concentrations therefore this research is limited to infer indirect associations between Mg^2+^ supplementation and performance from dietary intake determined from food diaries. The study duration and the nature of a consecutive 2 day protocol both consisting of a 40 Km time trial can be seen as to limit the potential for subject recruitment and therefore final number of participants recruited. The use of males and females within groups must also be noted to account for occasional group data variance, on various parameters and a high level of standard deviation.

## Conclusion

The current study showed a positive effect with A Mg^2+^ supplementation in relation to net strength and force gains with bench press, findings that support previous research [11,22,23,25]. Further, cardiovascular responses to the bench press were significantly enhanced by Mg^2+^ supplementation reducing resting SBP and DBP with the greatest effect seen with A Mg^2+^ supplementation for rest and post exercise. Similarly, SBP, DBP and Aix showed a significantly greater and more consistent reduction in response to the A Mg^2+^ loading strategy, as opposed to the minimalistic effect induced by Chr Mg^2+^ loading strategy.

In conclusion, it can be stated that improvements seen with the A loading strategy cannot to the same extent be observed with the Chr loading of Mg^2+^, thus potentially suggesting a regulatory effect within the body influenced by the duration of Mg^2+^ supplementation intake.

To conclude, from this study there appears to be no benefit in long term magnesium supplementation for those who have adequate dietary intake, but there are some benefits for taking an acute dose, particularly before intense exercise.

Future work may focus on the above parameters for those with low dietary Mg^2+^ intake and also for the optimum time that supplementation should be given to induce these positive findings.
