# Correction to: The effect of acute vs chronic magnesium supplementation on exercise and recovery on resistance exercise, blood pressure and total peripheral resistance on normotensive adults

**DOI:** 10.1186/s12970-018-0239-6

**Published:** 2018-07-25

**Authors:** L. S. Kass, F. Poeira

**Affiliations:** University of Hertfordshire, School of Life and Medical Science, College Lane, Hatfield, Hertfordshire, AL10 9AB UK

## Correction

The original article [1] contains an error whereby the datum value of ’17.7%’ in both the Abstract and the first line of the *Bench press* sub-section of the Results section is incorrect. The value should instead be ‘7.7%’.
